# MicroRNA expression profiles across blood and different tissues in cattle

**DOI:** 10.1038/sdata.2019.13

**Published:** 2019-02-12

**Authors:** Hui-Zeng Sun, Yanhong Chen, Le Luo Guan

**Affiliations:** 1 Department of Agricultural, Food and Nutritional Science, University of Alberta, Edmonton, AB, T6G 2P5, Canada

**Keywords:** RNA sequencing, Gene regulation, miRNAs

## Abstract

MicroRNAs (miRNAs) play essential roles in regulating gene expression involved in various biological functions. The knowledge of miRNA expression profiles across different tissues in cattle is still limited. Using the miRNAs data generated from 158 samples in three studies, we characterized the miRNA expression profiles of bovine sera, exosomes and 11 different tissues. Totally 639 miRNAs were identified and 159 miRNAs were expressed in all samples. After relative log expression normalization, four miRNA expression clusters were generated: 1) sera and exosomes; 2) liver; 3) mammary gland; 4) rumen and gut tissues. The top 10 most abundant miRNAs accounted for >55% of total miRNA expression in each tissue. In addition, this study described a detailed pipeline for identification of both tissue and circulating miRNAs, and the shareable datasets can be re-used by researchers to investigate miRNA-related biological questions in cattle. In addition, a web-based repository was developed, which enables researchers to access the distribution range and raw counts number of the miRNA expression data (https://www.cattleomics.com/micrornaome).

## Background & Summary

MicroRNAs (miRNAs) are endogenous short non-coding RNAs (typically contain 18-25 nucleotides (nt)) that play a vital role in regulating gene expression at the post-transcriptional level by inducing the degradation or blocking translation of target messenger RNA^1^. MicroRNAome refers to the complete set of miRNAs expressed in an organism/tissue and is an important component of feedomics, a recent proposed approach to study biological regulatory mechanisms that determine animal productivity, product quality, well-being and health using different omics technologies^2^. Animal miRNAs are highly conserved among different species, which target most of the protein-coding genes that are involved in many developmental and biological processes^3^. Bovine miRNA studies have proposed their significant roles in diverse molecular functions and biological processes including mammary gland development^4–6^, rumen development^7,8^, ovary development^9,10^, adipogenesis^11,12^, infection and immunity^13–15^, production traits improvement (e.g. milk yield and quality^16,17^), early diagnosis of pregnancy^18–20^ and so on. Therefore, study of the expression and distribution of miRNAs in various tissues is important for the better understanding of physiological and pathological mechanisms in cattle towards better strategies for sustainable production and animal health.

To date, the information of miRNAs expression profiles in different bovine tissues is still limited. Our lab has reported miRNA expression patterns in 11 tissues (brain, longissimus dorsi muscle, kidney, liver, spleen, thymus, cerebellum, medulla, hypothalamus, abdominal subcutaneous fat and rump subcutaneous fat) of beef cattle^21^, which has contributed to the first and up-to-now the most comprehensive analysis of miRNA abundance in different tissues of bovine species. However, the previous study mainly focused on the visceral tissues and lacked the expression profiles of the gastrointestinal tract and circulating miRNAs in body fluids (blood). Meanwhile, a relatively low number of miRNAs (n = 228) were identified due to the poor and low sensitivity of the sequencing technology used at the time. It is necessary to reveal the bovine miRNAs expression profiles across a wide range of tissues using more advanced techniques.

**Figure 1:**
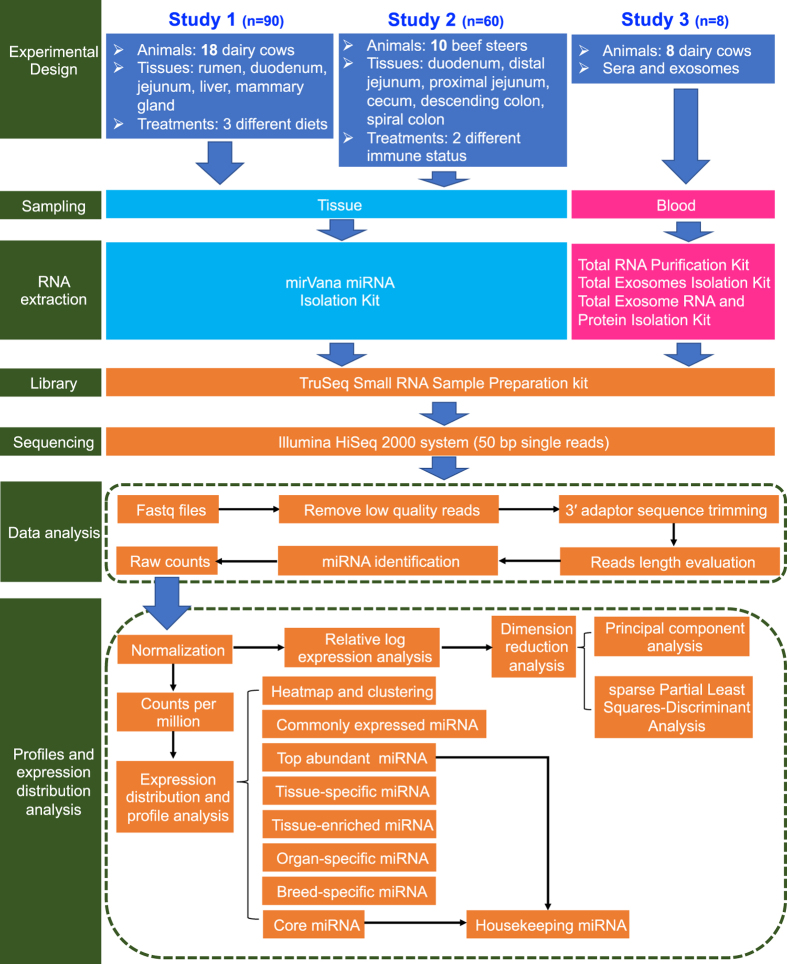
The workflow of collecting data and technical analysis in this study.

In this study, we analyzed the miRNAs data generated from 158 samples in our previous three different studies to improve the understanding of miRNA expression across bovine tissues and blood. The workflow of data collection and technical analysis is illustrated in Fig. 1. The 158 samples were derived from five different dairy cow tissues (study 1)^17^, six different beef cattle tissues (study 2)^22^, as well as dairy cow sera and exosomes (study 3)^23^, which were all used the same library construction method (TruSeq Small RNA Sample Preparation kit) and high-throughput sequencing platform (Illumina HiSeq 2000 system) to identify the miRNA expression profiles (Fig. 1). Exosomes are small extracellular vesicles (40–100 nm in diameter) derived from multivesicular body-sorting pathway and present in diverse body fluids, which contain various molecular constituents including proteins, mRNAs, miRNAs^24^. A detailed pipeline of generation and comparison of expression of miRNAs across various tissues and blood from different datasets is also provided (Fig. 1). The Gene Expression Omnibus (GEO) records of studies 1 and 2 were the first time to be released under this study (Data Citation 1 and Data Citation 2), and GEO record of study 3 has already been released with the previous publication (Data Citation 3).

**Figure 2:**
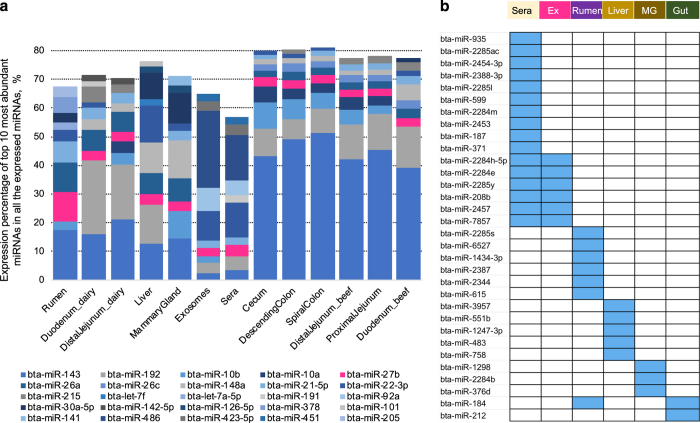
Top 10 highly expressed microRNAs (miRNAs) and uniquely expressed miRNAs in the bovine sera, exosomes and 11 different tissues. **a**. The top 10 miRNAs with the highest expression levels in the bovine sera, exosomes and 11 different tissues. The bta-miR-143 and bta-miR-27b are labeled in blue and pink respectively. **b**. The uniquely expressed miRNAs in the sera, exosomes, rumen, liver, mammary gland, and gut-related tissues. Ex = exosomes; MG = mammary gland.

A total of 639 miRNAs were identified, among which 436 miRNAs were expressed in at least one tissue, 202 miRNAs were expressed in all tissues, and 159 miRNAs were expressed in all samples (miRNA_profile, Data Citation 4). Totally 9, 3, 7, 5, 9, and 1 tissue-enriched miRNAs were identified in rumen, liver, mammary gland, sera, exosomes, and cecum, respectively (miRNA_profile, Data Citation 4). The commonly and uniquely expressed miRNAs between dairy cow and beef steers in duodenum and jejunum were identified and presented in the Figshare Database (miRNA_profile, Data Citation 4). The top 10 miRNAs with the most abundant aligned reads accounted for more than 55% of total miRNA abundance in sera, exosomes and each tissue (Fig. 2a). Among them, the expression of bta-miR-143 and bta-miR-27b was detected in all samples, which is expressed under both control and experimental conditions in each study (Fig. 2a), confirming that both miR-143 and miR-27b were highly conserved across vertebrates^25,26^. Tissue-specific miRNAs included six rumen-specific miRNAs: bta-miR-2285s, bta-miR-6527, bta-miR-1434-3p, bta-miR-2387, bta-miR-2344, and bta-miR-615; five liver-specific miRNAs: bta-miR-3957, bta-miR-551b, bta-miR-1247-3p, bta-miR-483, and bta-miR-758; three mammary gland-specific miRNAs: bta-miR-1298, bta-miR-2284b, and bta-miR-376d (Fig. 2b). In addition, expression of bta-miR-2284h-5p, bta-miR-2284e, bta-miR-2285y, bta-miR-208b, bta-miR-2457, and bta-miR-7857 were only detected in the circulating fluids (Fig. 2b) and ten specific miRNAs were only identified in sera: bta-miR-935, bta-miR-2285ac, bta-miR-2454-3p, bta-miR-2388-3p, bta-miR-2285l, bta-miR-599, bta-miR-2284m, bta-miR-2453, bta-miR-187, and bta-miR-371. The bta-miR-184 was rumen and gut tissue specific miRNA and bta-miR-212 was only expressed in lower gut tissues (Fig. 2b). The tissue-specific miRNAs can be used to investigate the unique expression patterns and functions in certain organs/tissues of interests.

This study describes the comprehensive datasets of multi-tissue miRNAome, which provides a valuable resource for bovine functional genomics research and can be re-used by the research community to address additional biological questions on miRNA expression profiles in cattle. The miRNA expression profiling workflow in this study included both tissue and circulating miRNAs that can be applied to human and the other livestock animal research. The information provides applicable clues for the understanding of the roles of miRNAs in tissue development and tissue-specific functions contributing animal production and health.

## Methods

The experimental procedures using dairy cows in study 1 were in accordance with the protocol approved by the Animal Care Committee, Zhejiang University, Hangzhou, P. R. China. All animals used in studies 2 and 3 were managed based on the guidelines established by the Canadian Council of Animal Care^27^ and the experimental procedures were approved by the University of Alberta Livestock Animal Care and Use Committee.

### Samples collection

Totally 150 samples from 11 different tissues and 8 circulating fluid samples in cattle were used in this study. For tissue samples, the rumen epithelium, duodenum, jejunum, liver, and mammary gland samples were collected from 18 mid-lactation Chinese Holstein dairy cows^17^, the duodenum, distal jejunum, proximal jejunum, cecum, spiral colon, and descending colon were collected from 10 British × Continental yearling feedlot beef steers^22^. For the circulating fluid samples, sera and exosomes in the whole blood were extracted from 4 healthy lactating Holstein cow^23^. Two 2 × cm^2^ samples were collected from each tissue after animal were slaughtered and were immediately frozen in liquid nitrogen within 10 min. The whole blood samples were processed with two runs of centrifugation to get the sera: 4 °C, 1,900 × g, 10 min for the first run and 4 °C, 16,000 × g, 10 min for the second run. Exosomes were isolated from the same sera samples using the Total Exosomes Isolation Kit (Invitrogen, Carlsbad, CA, USA). All the samples were stored at −80 °C for further analysis.

### RNA extraction

The frozen tissues were ground to a fine powder in liquid nitrogen before RNA extraction. Totally RNA was extracted from approximately 100 mg ground powder using the mirVana miRNA Isolation Kit (Ambion, Carlsbad, CA, USA) based on the manufacturer’s instruction. For the circulating samples, total RNA in sera was isolated using Total RNA Purification Kit (Norgen, Thorold, ON, Canada) and the total RNA in exosomes was extracted using Total Exosome RNA and Protein Isolation Kit (Invitrogen, Carlsbad, CA, USA) following the manufacturer’s protocols. The concentration of RNA was determined using the Qubit microRNA Assay Kit and the 2.0 Fluorometer (Invitrogen, Carlsbad, CA, USA). The RNA integrity number (RIN) was obtained using the Agilent 2100 Bioanalyzer (Agilent Technologies, CA, USA).

### miRNA-Seq library preparation and sequencing

About 1 μg of total RNA from each sample were used to construct small RNA library using the TruSeq Small RNA Sample Preparation kit (Illumina, San Diego, CA, USA) according to the manufacturer’s instructions which included ligating adapters to 3’ and 5’ end of the RNA molecules, reversely transcribing and amplifying libraries, purifying cDNA, and checking and normalizing libraries. PCR enrichment was performed with 11 cycles. Small RNA libraries were then pooled together in equal amounts for gel purification. All libraries were sequenced at Génome Québec (Montréal, Canada) using the HiSeq 2000 system (Illumina, San Diego, CA, USA) to generate 50 bp single reads.

Totally 11.50 ± 5.60, 15.14 ± 4.14, 11.95 ± 2.46, 9.53 ± 3.96, 11.24 ± 6.88, 3.00 ± 0.70, 3.77 ± 2.00, 4.11 ± 1.58, 3.99 ± 1.38, 7.90 ± 1.44, 3.82 ± 1.13, and 7.54 ± 7.23, 1.79 ± 0.25, 7.90 ± 1.44 million raw reads were generated for rumen, duodenum (dairy cow), jejunum (dairy cow), liver, mammary gland, duodenum (beef steer), distal jejunum (beef steer), proximal jejunum (beef steer), cecum, spiral colon, descending colon tissues, exosomes and sera, respectively (Raw_reads, Data Citation 4).

### Sequencing data analysis

Low quality miRNA reads in raw sequence data were removed using Illumina CASAVA (version 1.8) and the reads that passed quality filtering were subjected to sRNAbench (http://bioinfo5.ugr.es/srnatoolbox/srnabench/) for 3’ adaptor sequence trimming and reads length distribution analysis^28^. The sequences with read length larger than 15 nt were aligned to the corresponding miRNA precursor sequences in bovine miRNA database (miRBase, version 22) to identify known miRNAs using sRNAbench with the default parameters (alignment type: bowtie seed alignment; seed length for alignment: 20; minimum read count: 2; minimum read length: 15; allowed number of mismatches: 2; maximum number of multiple mappings: 10). The read counts of identified miRNA in each sample were generated and sorted out into one matrix.

### miRNA profiling comparison among different tissues

One of the main factors contributing to expression variation among tissues is batch effect resulting from different RNA-Seq experiments, which are the systematic non-biological differences between batches (experiments) of samples including PCR amplification and reverse transcription artifacts, systematically bad sequencing cycles and errors in base-calling^29^. To check the batch effect between the samples, we performed relative log expression (RLE) analysis using RUVSeq package in RStudio (version 1.0.136), which is defined as the log-ratio of a read count to the median count across samples for each miRNA. The variables with zero value in more than two-third samples were removed in the data filtering step. The 0 value left was simulated using half of the minimum value before log transformation. The upper-quartile normalization method in *betweenLaneNormalization* function of EDASeq was applied in raw count normalization^30^. The comparable samples should have similar RLE distributions that are centered around zero^31^.

The dimension reduction method, principal component analysis (PCA) was conducted using the RStudio (version 1.0.136). The sparse partial least squares-discriminant analysis (sPLS-DA) was further performed to produce robust and easy-to-interpret models by effectively reducing the number of variables^32^. The number of component and number of variables in each component were set at 5 and 10, respectively. In the heatmap, the top 30 of miRNAs ranked by ANOVA were used to show the most significant different patterns. Euclidean method and Ward algorithm were applied to measure distance and generate clustering respectively.

### Distribution of miRNA expression among different tissues

The expression level of miRNA was assessed using counts per million reads (CPM) with the following formula: CPM = (reads number of one miRNA) ÷ (total mapped reads to all annotated miRNAs) × 10^6^. Expressed miRNA was defined as a miRNA with CPM > 1 in one sample and tissue/organ-expressed miRNA was defined as a miRNA with CPM > 1 in more than 50% of samples in one tissue/organ. Tissue-specific miRNA was defined as a miRNA uniquely expressed in one tissue. Tissue-enriched miRNA was defined as miRNAs in one tissue type at least three times of the maximum levels of all other tissues. The bovine sera, exosomes and 11 tissues were classified into 6 organs: rumen, liver, mammary gland, small intestine (duodenum in dairy cow and beef steer, jejunum in dairy cow, distal and proximal jejunum in beef steer), large intestine (cecum, descending and spiral colon), and blood vessel (sera and exosomes). Organ-specific miRNA was defined as a miRNA uniquely expressed in one organ.

### Code availability

RStudio (version 1.0.136) was used for data reading, normalization, analysis and figure generation of RLE, PCA, sPLS-DA, and heatmap. The related codes are available in the Figshare Database (R_code, Data Citation 4).

## Data Records

All sequencing data have been uploaded to the National Center for Biotechnology Information (NCBI) Gene Expression Omnibus (GEO). Each dataset contains microRNA expression raw data files (fastq format), processed data files (raw counts of sequencing reads), and a metadata spreadsheet referring to the information about the overall study and individual samples. All data can be used without restrictions. Tables consisting of all the miRNA identified in bovine different tissues and blood and raw reads number in each sample were uploaded to the Figshare Database (Raw_reads, Data Citation 4).

## Technical Validation

The RNA concentration was assessed using the Qubit microRNA Assay Kit and the 2.0 Fluorometer (Invitrogen, Carlsbad, CA, USA). The quality of RNA was evaluated using the Agilent Bioanalyzer (Agilent Technologies, Santa Clara, CA, USA).

**Figure 3:**
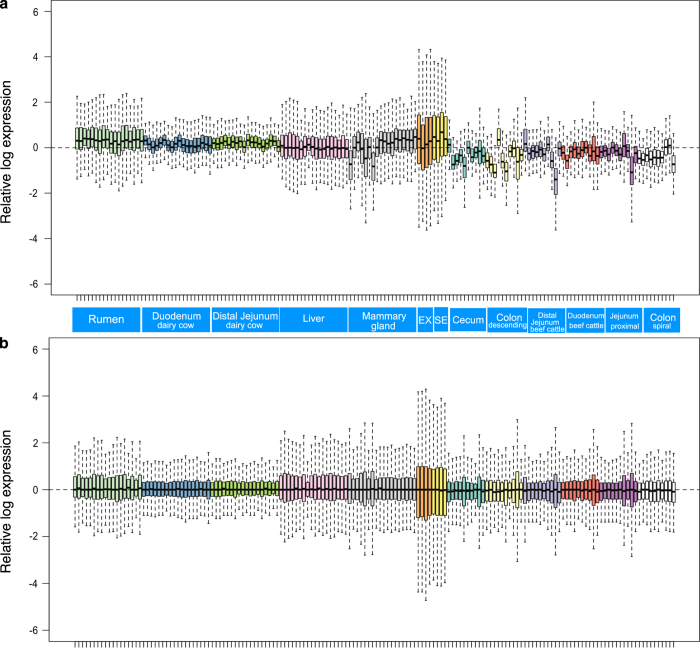
Relative log expression analysis (RLE) of 158 samples based on their microRNA raw counts data. Samples in different tissues were displayed in different colors. **a**. The Boxplots of un-normalized sample RLE. **b**. Boxplots of normalized sample RLE. EX = exosomes, SE = sera.

**Figure 4:**
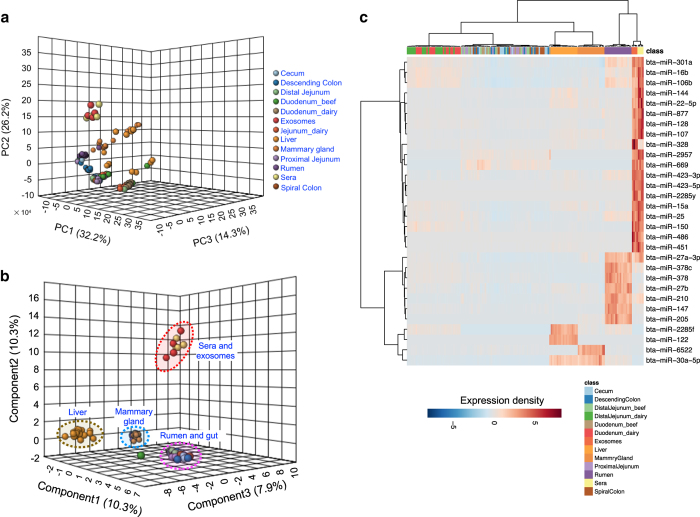
The discriminating profiles of microRNAs (miRNAs) expression in different tissues. **a**–**c** represent the 3-D PCA plot, 3-D sPLS-DA plot, and the heatmap of the miRNAs expression in the bovine sera, exosomes and 11 different tissues. Each tissue was displayed using different colors. PCA = principal component analysis; sPLS-DA = sparse partial least squares-discriminant analysis. The 11 tissues include dairy cow rumen, duodenum, jejunum, liver and mammary gland, beef cattle duodenum, distal jejunum, proximal jejunum, cecum, spiral colon and descending colon.

RNA samples with the RNA integrity number >7.0 and the ratio of 28 S/18 S ranging from 1.7 to 2.4 were used for small RNA library construction. The RIN numbers of all samples are listed in Figshare Database (RIN_table, Data Citation 4). The quality control of sequencing reads was performed using sRNAbench in sRNAtoolbox (http://bioinfo5.ugr.es/srnatoolbox) with the parameters of minimum adaptor length >10, minimum read count >2, and minimum read length >15. Comparable normalization was conducted using relative log expression analysis. As shown in Fig. 3a, the boxplots revealed the need for between-sample normalization. After the RLE normalization of miRNA read counts, the 158 samples in bovine sera, exosomes, and 11 different tissues were centered around zero roughly (Fig. 3b), which represent ideal appearance of batch effect elimination^33^. The miRNA expression profiles of sera and exosomes were mostly separated from those of other tissues in the 3-D PCA plot, meanwhile, those of mammary gland, rumen, and liver were also separated from lower gut tissues (Fig. 4a). The miRNA expression profiles in these gut tissues including dairy cow duodenum and jejunum, beef cattle duodenum, distal jejunum, proximal jejunum, cecum, spiral colon and descending colon didn’t show obvious discriminations (Fig. 4a). The sPLS-DA plot displayed four significantly different clusters based on overall miRNA expression profiles: 1) sera and exosomes; 2) liver; 3) mammary gland; 4) rumen and gut tissues (Fig. 4b). The individual samples in different gut tissues were grouped in one cluster rather than clustering within corresponding tissues in the heatmap with the top 30 most different expressed miRNAs (Fig. 4c).

## Additional information

**How to cite this article**: Sun, H.-Z. *et al*. MicroRNA expression profiles across blood and different tissues in cattle. *Sci. Data*. 6:190013 https://doi.org/10.1038/sdata.2019.13 (2019).

**Publisher’s note**: Springer Nature remains neutral with regard to jurisdictional claims in published maps and institutional affiliations.

## Supplementary Material
